# Malnutrition Rates in Chile from the Nitrate Era to the 1990s

**DOI:** 10.3390/ijerph182413112

**Published:** 2021-12-12

**Authors:** Manuel Llorca-Jaña, Diego Barría Traverso, Diego del Barrio Vásquez, Javier Rivas

**Affiliations:** Escuela de Administración Pública, Facultad de Ciencias Económicas y Administrativas, Universidad de Valparaíso, Valparaíso 2362415, Chile; diego.barria@uv.cl (D.B.T.); diego.delbarrio@uv.cl (D.d.B.V.); javier.rivas@uv.cl (J.R.)

**Keywords:** malnutrition, Chile, stunting rates, anthropometry, height

## Abstract

Following Salvatore and the WHO, in this article, we provide the first long-term estimates of malnutrition rates for Chile per birth cohort, measured through stunting rates of adult males born from the 1870s to the 1990s. We used a large sample of military records, representative of the whole Chilean population, totalling over 38 thousand individuals. Our data suggest that stunting rates were very high for those born between the last three decades of the nineteenth century and the first two decades of the twentieth century. In addition, stunting rates increased from the 1870s to the 1900s. Thereafter, there was a clear downward trend in stunting rates (despite some fluctuations), reaching low levels of malnutrition, in particular, from the 1960s (although these are high if compared to developed countries). The continuous decrease in stunting rates from the 1910s was mainly due to a combination of factors, the importance of which varied over time, namely: Improved health (i.e., sharp decline in infant mortality rates during the whole period); increased energy consumption (from the 1930s onwards, but most importantly during the 1990s); a decline in poverty rates (in particular, between the 1930s and the 1970s); and a reduction in child labour (although we are less able to quantify this).

## 1. Introduction

In Salvatore’s [1] recent study, published in this journal, a general-purpose methodology recommended by the World Health Organization (WHO) [2,3,4] was applied to estimate malnutrition rates. According to the WHO [2,3,4], malnutrition refers to deficiencies, excesses or imbalances in a person’s intake of energy and/or nutrients. Of these three conditions (i.e., undernutrition, micronutrient-related malnutrition, and overweight), we are only dealing with undernutrition, and in particular, with wasting, measured as stunting (low height-for-age).

Following Salvatore’s methodology, and with the aim of testing it in new case studies, in this article we estimate malnutrition rates for Chilean males born from the 1870s to the 1990s, using a large adult height sample of military records taken from Llorca-Jaña et al. [5,6], and which is representative of the whole Chilean male population. These are indirect childhood malnutrition estimates based on male adult height, working under the reasonable assumption that if the nutritional status of a child is inadequate, this child will reach an adult height far lower than under more favourable circumstances, ceteris paribus [7]. Inadequate physical growth (e.g., measured through height) will result in both children and adults who are stunted or short if compared with a widely accepted standard [7], either national or international [1].

The main aim of this article is to provide the first long-run estimates of male’s malnutrition rates in Chile for cohorts per decade of birth at a national level, thus making an important contribution to the literature on Chilean living standards. By adding new estimates of malnutrition, we are providing a different aspect of the evolution of living standards in Chile from the nitrate era. We are also adding evidence for those interested purely in economic growth, since those who escape malnutrition can join the labour force [7]. In addition, we provide a set of explanations to account for the long-term decline of malnutrition in Chile. Therefore, this contributes to the study of the biological welfare of a developing country in historical perspective, and in particular, of lower socio-economic groups. Most, if not all, of those who suffer stunting belong to the lower strata of society, and are typically invisibilised by the historiography. Moreover, we provide a comprehensive review of the main public policies implemented by a developing country to diminish malnutrition and to improve the overall biological welfare of its population.

Standards of living should not be measured only by economic variables, and the more variables we have on biological welfare, the better [7]. For example, better nutrition is linked to improvements in cognition and in labour productivity. This information is not captured by conventional indicators, such as per capita GDP, and it is important to take a multidimensional approach to the study of living standards [7]. Most studies concerned with malnutrition in Chile focus on specific periods, rather than cultivating a long-term analysis (e.g., [8,9,10,11,12,13,14,15,16,17,18]). Furthermore, most of the extant studies on nutrition for this country, and which provide proxies of malnutrition rates, concentrate on recent decades (e.g., [19,20,21,22]). Fernando Monckeberg [23], an authority on this subject, went as far as to declare, in 1977, that the only serious attempt to estimate undernutrition in Chile (at a national level) was a study conducted as late as 1969. There is little data for cohorts born before the mid-twentieth century.

This gap is of consequence since anthropometric measurements, such as height, body mass index, and birth weight, all influenced by populational nutritional status, have gained importance as indicators of standards of living which adequately capture measures of inequality [7]. We know little regarding how some of these phenomena operate in Chile and have scant data with which to track changes in food supply and consumption patterns. This paper seeks to improve our understanding of the evolution of the nutritional status of the lower strata of the Chilean population for those born between the 1870s and the 1990s, and in particular, the relevance of biological welfare.

One recent and valuable study has been provided by Núñez and Pérez [24]. However, the samples used by these authors have several shortcomings or differences with our methodology. Most of the data used by Núñez and Pérez relate to urban settings, in particular for the capital of the country (Santiago), while our data is representative of the entire country. Their data consists of 1.258 height-for-age (HAZ) Z scores for the selected (aggregated) samples, rather than individual observations of the height per person, as we use here, in order that they cannot observe individual variations in height-for-age within the samples (as they have acknowledged, p. 11). In addition, their sample data for the nineteenth century is rather small (indeed, they grouped their results for the 1870s–1900s as a whole, rather than providing data per decades, as we do here). More importantly, it underrepresents people from a lower socioeconomic status (SES), in particular, during the earliest period of their study. The main focus of Núñez and Pérez is the analysis of differences in height-for-age for different SES, a topic in which they made their greatest contribution. Despite these shortcomings, the study by Núñez and Pérez [24] is a welcome contribution, and our conclusions are similar in some respects.

Moreover, we have contrasted our new malnutrition estimates with newly available information on mortality rates [25] (as better nutrition and better health lowers mortality by improving resistance to diseases [26]), and on per capita consumption of both energy and key foodstuffs in Chile from the 1930s [27,28], which is also available per decade of birth. At this point, it is important to mention that stunting is not only the result of a poor diet, but it also depends on the existence of other demands on the body’s energy supplies, such as childhood disease or child labour [7]. It is rarely possible to ascribe a single cause to changes in stunting rates, thus the importance of considering other variables, such as infant mortality rates and child labour regimens [1].

Our estimates show that male malnutrition rates were very high, and on the increase, for those born between the 1870s and the 1900s (in line with the anthropometric data of Llorca-Jaña et al. [5,29], and fitting with Fogel’s [30] idea of the prevalence of chronic malnutrition in many countries until the first decades of the twentieth century). Thereafter, there was a continuous decline, until the 1930s (this finding differs from that of Núñez and Pérez, whose data shows stagnation in malnutrition rates for this period). In addition, there was a new downward trend from the 1940s to the 1990s (except for the convoluted 1970s). Moreover, there was a downward trend of moderate and severe malnutrition from the 1910s to the 1990s, despite some mild fluctuations (also present in Salvatore’s pioneer study for Argentina).

Our estimates show that Chile’s (moderate and severe) malnutrition rates declined from 15–19% for those born during the 1870s–1900s to some 2% for those born during the 1990s, a remarkable achievement for a middle-income country. This dramatic fall in malnutrition rates is important since it is well known that when this happens, in any country, ceteris paribus, there is an increase in cognition and labour productivity (thus in economic growth), a reduction in mortality and a decrease in the prevalence of infectious diseases, leading to an increase in life expectancy: Stunting rates are effective predictors of the risk of morbidity and mortality [7,30,31,32,33,34]. Furthermore, as highlighted by Fogel [30], individuals who are stunted, even if they are otherwise healthy during adulthood, will be at an increased risk of dying prematurely (see also [7,35]). Finally, Deaton ([32], p. 43) has noticed that Chile has “as good life expectancy as the United States, at about a quarter of per capita income and about 12 percent of per capita health expenditures”, in order that this fall in malnutrition may explain part of this story.

## 2. Methodology

The two standard anthropometric measures of malnutrition rates are height-for-age and weight-for-age, usually used for children (in particular, during early infancy), but in our case applied to adult heights. There is no long-term series of malnutrition estimates based on weight-for-age data for Chile (weight data is scarce for any period before the 1990s), thus we need, necessarily, to resort to height-for-age information. It is believed that a low height-for-age is a result of stunting or in other words, a retardation of “normal” growth [30]. Height-for-age is regarded as the best indicator of stunting, since height captures adequately deficiencies in nutrition and health insults during infancy, as well as the prevalence of child labour [1,30,32].

**Figure 1:**
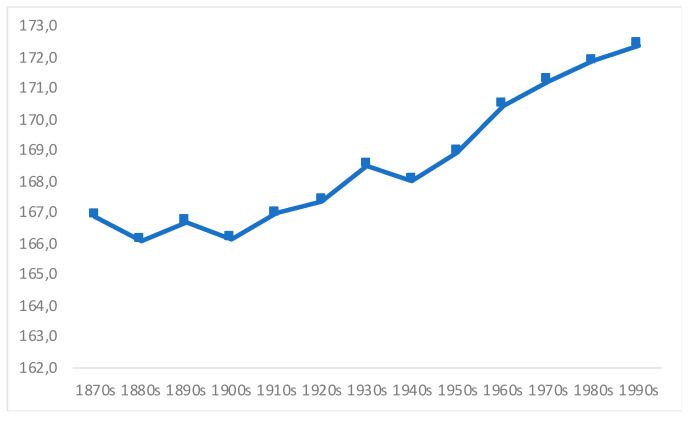
Raw average height of Chilean male adults (cms). Source [5,6].

Following Salvatore’s [1] study for Argentina, we have estimated Chile’s malnutrition rates based on the distributions of adult height of men born from the 1870s to the 1990s, using a large military sample (see below), which is representative of the whole country [5,6], whose raw averages are shown in Figure 1. This nationwide representation is an important contribution to the current scholarship. In addition, while the first direct estimates on Chileans’ diet (energy consumption) come from the 1930s [28], we are providing indirect estimates from the 1870s, adding some 60 years of new evidence.

The physical stature of these adults was a direct result of their nutritional status during childhood, and that is why we provide the data per decade of birth, a usual procedure in anthropometric history. Malnutrition during childhood is believed to be the result of many factors apart from insufficient nutrition: Diseases, environmental insults, and excessive (physical) labour [7,30,32,33,34].

We have contrasted our data against the boys’ growth standards from the World Health Organization, which have been published from 1983 [1,2]. Similar to Salvatore [1], we opted for the WHO tables as a benchmark since these WHO distributions are based on large samples from a wide array of countries, and also since they can be used for comparison with other studies: It is a universal standard. Another study for Chile that also used the WHO as a benchmark is that of Ivanovic [36], but this covered a short period of time. An alternative, used by Núñez and Pérez [24], would be to compare our data against growth curves in the USA, in particular, the data produced by the US Centre for Disease Control and Prevention, CDC, or against a widely accepted national growth curve, unavailable for Chile (although it is available for other Latin American countries, such as Argentina, see Salvatore [1]). The only growth tables available for Chile are those of Patri et al. [37]. However, they covered the age range from 0 to 6 years, thus they were of little use for our methodology (which requires tables up to 18 years, see below). In addition, Avendaño and Valenzuela [38], who did cover later ages, based their study on recent evidence of children in north Santiago rather than at a national level (for a discussion of Chilean children’s national growth tables, see Youlton and Valnezuela [39]). Despite the important contribution of these studies, they were not widely adopted as a benchmark at a national level by Chile’s health authorities.

In 1994, only six years after the publication of Avendaño and Valenzuela, the Chilean health service started to officially use the CDC standards to evaluate the growth of Chilean children, changing to the WHO tables in 2007 [40,41]. In turn, the CDC tables had replaced the French Sempé (Centre International de L’ Enfance) standards in use from the 1970s until 1994 [42,43,44]. The Sempé standards were widely used before the mid-1990s as there were no WHO tables, and also due to the belief that French growth tables were the most comparable to Chile [45]. Before Sempé’s tables, those used by paediatricians in Chile were mainly based on US tables [46] or British tables [47], thus always based on foreign information [39].

We agree with Salvatore [1] that none of these standards is better than the others, while also believing that it would be advisable to use those of the WHO to promote a comparison across Latin American countries, as we do in this article. There are some differences between the WHO and CDC charts (or Sempe´s charts) by age group. However, these differences are particularly important during early infancy, rather than at 18 years [48], in order that these differences are of less consequence for our methodology.

According to the latest WHO height-by-age distribution [4], a normal, well-fed, and healthy 18-year-old male at the 50th percentile should measure 176.1 cm with a standard deviation (SD) of 7.47. Following Salvatore [1], we have considered as stunted in our sample all of the observations (head count) that fall to the left of the WHO benchmark of 2 SDs from the mean (or the 50th WHO standard). The sample includes estimates from moderate (2 SDs) to severe malnutrition (over 3 SDs), which is a standard statistical cut-off point for stunting for undernourishment [24,34,41,49,50,51,52,53].

Similar to Salvatore, we noted that this distribution of stature for 18-year-old boys is similar to the adults, since most of the physical growth takes place from birth to the age of 18. Prolonged malnutrition during childhood and adolescence leads to adult stunting [7]. Therefore, the age range of our sample was 18–55, since it is only from the age of 55 that people start shrinking in height [54], although most of the data are for men in their 20s and 30s. Finally, we compared our data against the WHO benchmark of 2 SDs from the mean to estimate the stunting rates in Chile for men born from the 1870s to the 1990s.

Stunting rates calculated from adult populations (Salvatore’s methodology and our own estimates) could be lower than stunting rates estimated from data on children, as provided by Núñez and Pérez. They are not entirely comparable, but the long-term trends of the series produced by these alternative methodologies should show similar patterns, and this is why we have included here a comparison with the recent series of Núñez and Pérez.

Finally, our methodology is based on information for men only: Ideally, we would have also provided data for women, as we intend to do in future research projects. A larger dataset would also allow us to provide data per regions of birth.

## 3. Data Sources

We have resorted to a large database of military records used by Llorca-Jaña et al. [5,6], totalling 38.308 observations extracted from nearly 3.000 loose volumes from the *Archivo Histórico del Ejército* (Army’s Historical Archive). It includes adult males born across the whole of Chile, including all of the provinces. We decided to exclude soldiers aged 17, to follow Salvatore [1] as closely as possible. In addition, we excluded those born before the 1870s due to a low number of observations per decade before that period. A great advantage of these data is that for the entire period considered by this article the height data stemmed from general conscription. Therefore, no selectivity issues were found within the sample.

Equally important, there was no significant minimum height requirement (MHR) before the measurement: There is not any truncation to the left. A visual inspection of the data shows that there are many observations below these referential MHR, and that there was no particular truncation at either 155 or 160 cm. The histogram of the data shows a quasi-normal distribution. More importantly for us, these MHR values fall below the WHO benchmark of 2 SDs from the mean. The data are representative of the entire Chilean population at that time, per socio-economic groups (including literacy rates and occupations) and per region of birth. All of the men included in our database were born in Chile, distributed across the whole of the country, and in a fairly representative fashion regarding the actual distribution of the national censuses of the time [5,6]. For the nineteenth century, the average raw height was 166.7 cm, with a SD of 6.1, while for the twentieth century data, the average was 168.9 cm and a SD of 5.9. Further details regarding the nature of the data can be found in Llorca-Jaña et al. [5,6].

## 4. Results: Estimates of Stunting Rates

**Figure 2:**
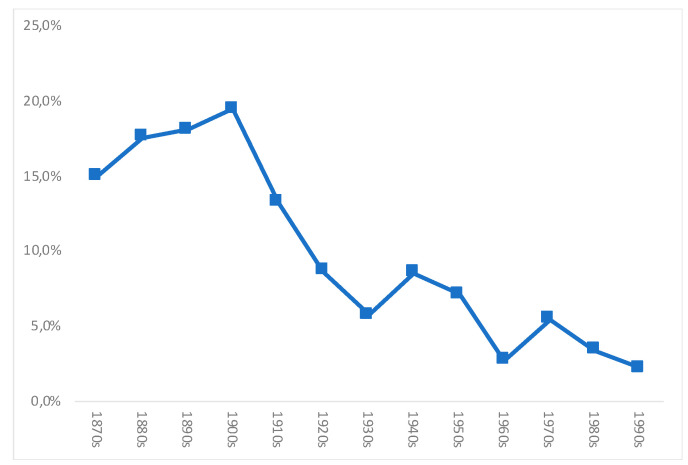
Evolution of moderate and severe stunting in Chile (as a share of male population, %), per decade of birth. Source: Table 1.

**Figure A1:**
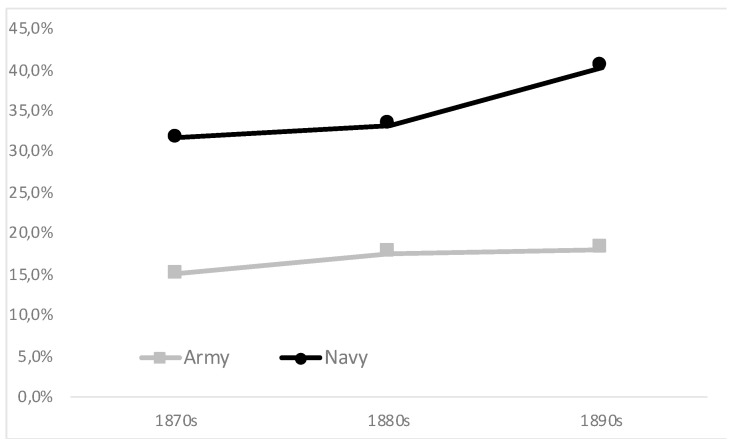
Source: Figure 2 for army records, Llorca-Jaña et al. [29] for navy records. **Note 1:** The navy sample for those born from the 1870s to the 1890s include 28.431 observations, for marines aged 18 to 55. The average raw height is 164.1 cm and the SD is 6.2 cm, while the comparable values for the army sample are 166.7 and 6.1 cm, respectively. **Note 2:** The estimates based on naval records are far higher than those of the army due to some restrictions imposed by the Chilean navy, including some health requirements. Marines tend to be shorter than soldiers in the army, since navies around the world had lower minimum height requirements than the army: Tall navy recruits were usually excluded from navies given the limited size of ships’ cabins. There are fewer men of exceptional height than might be expected for a normal distribution [29].

Following Salvatore [1], we estimated stunting rates for Chile (i.e., moderate and severe malnutrition rates), per decade of birth, according to the WHO 2007 standard. The results are shown in Table 1 and Figure 2. Stunting rates were high between the 1870s and the 1900s (as also found by Núñez and Pérez [24] with an alternative method), and increasing (alternative data based on navy records also show an increase for this period: See Figure A1 in Appendix A). On average, for the whole period 1870s–1900s, our stunting rate is 19%, while that of Núñez and Perez is higher, around 26%. Thereafter, there is a gradual but sustained decline until the 1930s, according to our estimates. This finding highlights an initial difference from Núñez and Pérez [24], for whom stunting rates remained at even higher levels during the 1910s–1930s (around 30%), in relation to their estimates for the 1870s–1900s (26%).

Although there is clearly a downward long-term trend from the 1900s to the 1990s, our series showed a mild increase in stunting rates for those born in the 1940s, if compared to the 1930s, but a renewed decline during the 1950s and 1960s, followed by another mild increase in the 1970s, and then further decreases during the 1980s and 1990s, when our series ends. The low level of our stunting rates estimates for the 1960s–1990s are in line with the data of Núñez and Pérez [24], but there are some important differences. Núñez and Pérez’s 2021 [24] estimates show a dramatic decline during the 1950s, and then again during the 1960s. Their stunting rates estimates for the 1950s are a third of the level of the previous decade (1940s), while the level of the 1960s is one-ninth of the achieved estimates in the previous decade (1950s). More importantly, by the 1970s, according to Núñez and Pérez’s estimates, moderate and severe stunting had almost disappeared from the country, while our estimates show that during the 1960s–1980s, despite an evident and important decline in stunting rates, malnutrition was by no means eradicated in Chile.

In support of our estimates, according to Monckeberg [21], total malnutrition rates among children younger than 6 years, although falling dramatically during the 1950s–1990s, were still at 37% in 1960, 19.3% in 1970, 11.5% in 1980, and 8% in the late 1980s, by no means negligible rates. In 1990, for children under 6 years, the rate was estimated at 7.4% [52] and by 2000 at 2.9% [21]. If only moderate and severe malnutrition is considered (as in Figure 2), the shares for children under 6 years are 6%, 3.5%, 1.6%, and 0.3% for 1960, 1970, 1980, and 1990, respectively, in line with our own estimates. It would be safer to put the 1990s (rather than the 1960s or the 1970s) as a turning point in the almost complete eradication of moderate and severe malnutrition in Chile, as was concluded by Yáñez [17] from qualitative evidence. That decade would also signal the emergence of another nutrition related issue in Chile: Obesity, a common feature of many developing countries during the last few decades [34], although the topic lies beyond the aims and ambitions of this paper.

## 5. Discussion of Main Findings

Regarding our estimates of stunting rates shown in Figure 2, the first finding that needs discussion is the high level of moderate and severe stunting rates we found for those born during the 1870s–1900s, 19% for these decades (on average), and up to the 1910s (despite the improvement of the last decade). These high rates would be consistent with, among other factors, high infant mortality rates (it is well known that malnutrition makes infections more severe, thus impacting on mortality rates [30]), and poor sanitary conditions more generally; poor nutritional status; and generalized child labour [1,7]. The high rates support the idea that malnutrition in Chile during this period was responsible for a large amount of the high general mortality suffered by the country.

**Figure 3:**
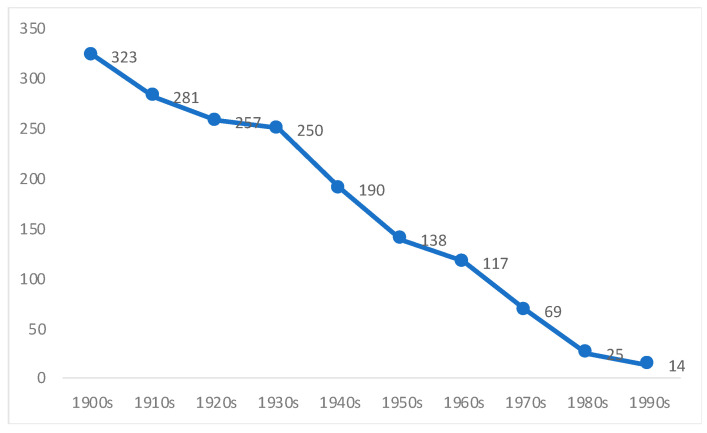
Infant mortality rates in Chile (boys only), 1900s–1990s (number of infant deaths for every 1000 live births). Source: Own elaboration from Llorca-Jaña et al. [25].

As seen in Figure 3, infant mortality rates for boys under 1 year of age were very high in Chile during these decades (the earliest available data are for the first two decades of the twentieth century). There was little public health expenditure during this period: Most of the health care was in the hands of the private sector and charitable institutions [20,55]. We have no data on the nutritional status for these early decades, but qualitative evidence does confirm that the diet of most Chileans was poor, and in particular, that it lacked animal proteins and dairy products [27]. Some contemporary works show a very low intake of energy consumption, and a diet dominated by wheat products [5,20,56,57,58]. The first available estimates of per capita consumption of meats and dairy products are for the 1930s, and they show very low levels for Chile [27,28]. The low level of education of the Chilean population at that time was detrimental to better food habits [20,53]. Generalized child labour is also well documented for this period: It was widely spread and socially accepted within mining companies (e.g., coal, nitrate), agriculture, the industrial sector, and urban settings: It was a part and parcel of the Chilean labour market [59,60,61]. Most of these activities clearly necessitated considerable energy consumption by these children, to the detriment of their physical growth.

**Figure 4:**
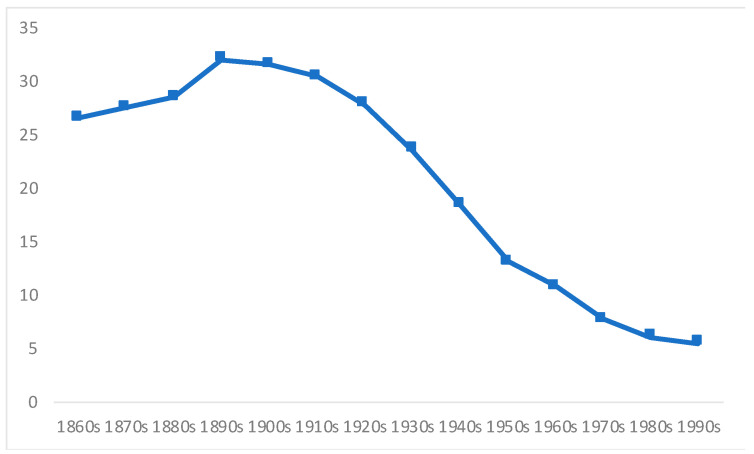
General mortality rates in Chile, 1860s–1990s (deaths per 1000 inhabitants). Source: Own elaboration from Díaz et al. [62] and Llorca-Jaña et al. [25].

A second finding that calls for an explanation is the increase in stunting rates from the 1870s to the 1900s, a period for which we have no infant mortality records. Yet, surprising as it may be (given booming exports and high economic growth), this fact is consistent with the increasing general mortality in Chile during the last decades of the nineteenth century (Figure 4), a large amount of which was caused by infants’ deaths, as well as by a stagnation in the biological welfare of the population, measured through adult height, towards the end of the nineteenth century. This was mainly due to the increasing urbanization combined with poor health and sanitation deprivation; increasing income inequality; and a deterioration in the epidemiological environment [5]. It is estimated that the intake of animal proteins during the late nineteenth century and early twentieth century was less than half of the equivalent level during the late colonial era, at least for the Central Valley [6]. Given the increase in general mortality rates, and the stagnation of average height, it comes as no surprise that stunting rates would increase between the 1870s and the 1900s, as shown by our data.

Thirdly, we need to explain the falling stunting rates from the 1900s to the 1930s, in particular, since we are at odds here with Núñez and Pérez [24]. Their data showed stagnation (even a mild increase) rather than a decline for this particular period. The decrease shown by our estimates is consistent with a pronounced fall during these decades in both the infant and general mortality in Chile (Figure 3 and Figure 4), and with the beginning of the implementation of social policies to improve health and education [55]. Unfortunately, we have no data on the per capita consumption of key foodstuffs during this period, but adult height increased continuously if slowly between the 1900s and the 1930s [6]. Furthermore, it is hard to believe that infant mortality declined without some improvements in the diet of the poorest strata of society [21]. From the mid-1920s, there was increasing public expenditure on health services, in both total and per capita terms [20,55]. Many laws were also promulgated from the mid-1920s to protect children and their mothers [23]. It was during the early twentieth century that the first critical voices were raised in opposition to the hardest sorts of child labour that emerged in Chile, which led to a decline in the most physically (and visibly) demanding sectors [59,60,63,64], especially the mining and the industrial sectors. For example, the mining code of 1874 established that children younger than 12 could not work in mining activities, but this regulation was largely ignored until 1919 [65]. In 1925, the government created the Superior Council for Infancy Protection, which was the predecessor of the Children Defence Council (1934) and the General Directorate of Infancy Protection of 1942 [64].

Next, and despite a mild increase in stunting rates during the 1940s (in comparison with the 1930s), it is clear that there is another marked downward trend between the 1940s and the 1990s (a continuation of the decreasing trend which started in the 1900s), as was also suggested by the data of Núñez and Pérez. Before entering into this long-term declining trend in stunting rates, it is useful to refer to the mild increase in stunting rates during the 1940s shown by our data. This could be explained in terms of a fall in the national per capita agricultural production, combined with inflation and a deterioration of real wages, which could have impacted on the nutritional status of the population. During this decade social conflict escalated, with an unprecedented number of strikes [66].

The downward trend which started in the 1910s and continued up to the 1990s is in line with continuous improvements in both the infant and general mortality in the country (Figure 3 and Figure 4), with increasing public expenditure on the provision of health services for most of the period [20,55], and with the institutionalization of public health including initiatives to combat malnutrition. Among the most important were, the promulgation of the first sanitary code in 1918 (leading to the creation of the General Sanitary Directorate), which was reformed in 1925 and monitored the quality of foodstuffs sold in the country [67]; the creation in 1937 of the National Feeding Council [68]; the creation of the National Health Service in 1952 (merging many institutions created from the mid-1920s, including the General Directorate of Infancy Protection [64]). We agree with Núñez and Pérez on the importance of the steady increase in public social expenditure, in particular, on education, health, the provision of supplemental feeding programs (food at schools, e.g., breakfast and lunches, one of the most common government interventions to prevent stunting anywhere [33]), public programs to deliver milk to deprived families, and the advent of a welfare state in Chile from the 1930s until 1973 (see also [60,66,69,70]).

**Figure 5:**
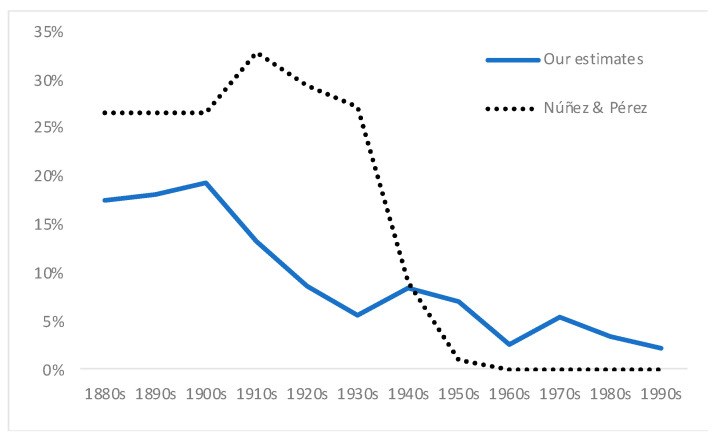
Evolution of moderate and severe stunting in Chile: Our data against Núñez and Perez’s estimates. Source: For Núñez and Pérez [24], for our estimates, Figure 2.

To support these schemes, 1937 saw the passing of the law known as “Mother and son”, whose benefits were perceived by a continuously larger share of Chilean children during the 1940s and 1950s, ensuring the provision of some basic medicines and foodstuffs [23,66,68,71,72]. In 1954, the Complementary Feeding National Scheme (PNAC in Spanish) was also launched [68,73]. Likewise, 1957 saw the creation of the National School Council, predecessor of the National School and Scholarship Assistance Council (JUNAEB) of 1964, which became the most important institution to ensure the distribution of food at schools, envisaged by the PNAC [60]. A mention should also be made of the emergence of social workers in Chile, whose duties included providing education on feeding habits, as well as on childcare and health practices, including those aimed at improving children’s nutrition [74]. The governments from the late 1930s also advocated for a better nutritional education across the population [75]. New legislation was passed to enrich wheat flour with vitamins and minerals, given the high per capita consumption of bread in the country [23,71]. The continuous decline in stunting rates is also in line with an improvement in the Chilean diet. Figure 5 shows that the per capita energy consumption did increase decade by decade from the 1930s (although starting from a low base, and remaining low by international standards for some time), except for a mild decline during the 1980s.

One marked difference between our estimates and those of Núñez and Pérez [24] is that for them there is a sharp structural break in the 1940s, which they justified mainly in terms of a rapid reduction in infant mortality. According to their data, stunting rates did not decline dramatically until the 1940s, while our data shows that the downward trend (of both mortality and malnutrition) started some decades earlier. As we have shown, there was also a sharp fall in infant mortality rates during the 1910s and 1920s, in order that it would be difficult to reconcile this fact with the idea of a structural break in the 1940s (Figure 5).

An important factor behind the fall in stunting rates from the 1940s is milk consumption, which increased significantly within the low SES, in particular, during the 1950s and 1960s, in part due to the above-mentioned government policies to promote milk consumption in schools [23,26,70,73,76], as well as the extension of the maternity leave period to promote breastfeeding [53]. Improvements in education and the increasing school enrolment rates [77], also meant that child labour diminished in the country, in particular, from the 1940s, at least in the industrial and mining sectors, where new legislation was passed to reduce it. There were a larger number of children at schools, where they could benefit from the government feeding programmes (i.e., most of the government food was given at public schools and/or medical facilities). Furthermore, increasing schooling delayed the entrance of children to the job market, at least for a few years [60]. New campaigns emerged against child labour in a wider range of economic activities, not only questioning the children’s working conditions, but also the fact that children had to work at all [63]. However, the impact of better education on improved nutrition was not only restricted to schools. From the mid-1950s the most important Chilean universities created their own nutritional research units, including the University of Chile’s INTA [68,78].

Our very low estimate of stunting rates in the 1960s is further supported by Moreno [20], who has shown that it was during the 1960s that the nutritional status of the lower ranks of society improved the most, thanks to the effective public policy, combined with an increase in real wages (see also Matus and Reyes [79]), in particular, of unskilled workers. The fall in infant mortality rates in Chile during the 1960s, 1970s, and 1980s, has been praised as one of the sharpest declines in the world, despite the low economic growth and high-income inequality [26]. Even when social expenditure decreased, it was focalised in the lower strata of society, ensuring that nutrition did not deteriorate for this SES [53].

We have stressed elsewhere that during the 1940s–1960s real wages increased. In addition, there were marked improvements in the national coverage of drinking water services, sewerage facilities, and electricity, up to the 1990s [6,26,80]. From the 1960s, there was an important decline in fertility rates in Chile: Families had fewer children in order that they could be better fed, thus improving their nutrition ceteris paribus [26]. The mild increase of stunting shown by our estimates during the 1970s, mainly under Pinochet’s dictatorship, is supported by the data provided by Moreno [20], showing a deterioration in the food consumption of families of low SES, together with soaring poverty [26] and the prevalence of child labour [60]. The data from the national census of 1970 suggest that even then, around 10% of children aged 12–14 worked in a regular fashion.

Another important difference between our estimates and those of Núñez and Pérez is that for them, stunting rates are (implicitly) close to zero for those born from the 1960s onwards, while our estimates for those born during the 1960s, 1970s, and 1980s are 3%, 5%, and 3%, respectively. Our data would suggest that there was some room for further improvement in the nutritional status of the population during these decades, as well as in health provision. In 1960, the first national survey on malnutrition was undertaken, by the US Interdepartmental Committee of Nutrition for National Defence. It concluded that the diet of Chilean children was still insufficient [60]. In the same vein, a report produced by the Chilean Agriculture Ministry in the late 1960s concluded that there were severe nutritional deficiencies across the Chilean population [81]. The 1970 presidential election campaign was characterised by all candidates offering specific nutrition schemes [26], which would not have been promised had the whole population been well nourished. In 1970, the Nutrition Commission of the Health Ministry recommended the creation of a Nutrition National Office to tackle undernutrition in Chile [23]. Yet, despite some advances in labour regulation, child labour persisted in Chile during the 1960s–1970s. It was not eradicated during these decades despite the welfare state that operated until 1973. For example, it is estimated that by 1960 around 17% of children aged 12–14 worked, and although this rate declined to less than 9% in 1970, it was still high by international standards [60].

**Figure 6:**
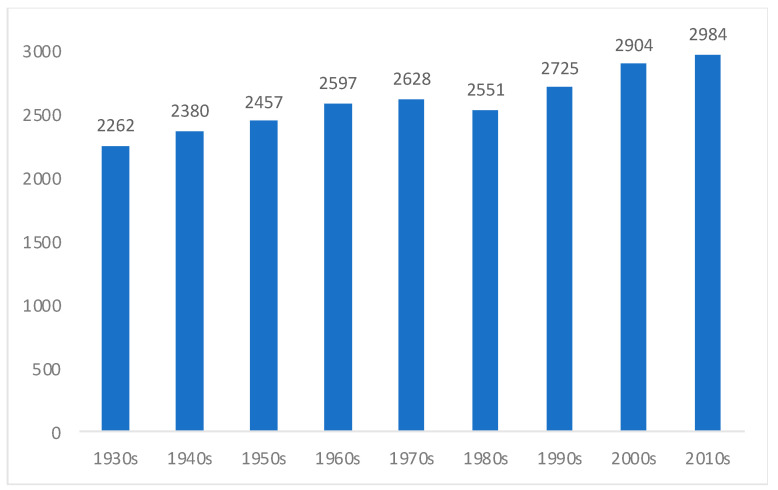
Chile dietary energy consumption (kcal per person per day), averages per decade, 1930s–2010s. Source: Own elaboration from Llorca-Jaña et al. [28]; and Solimano and Hakim [70]. Note: FAO online data started in 1960. Data for the previous decades were collected from printed FAO sources (detailed in Llorca-Jaña et al. [28]). Before the 1930s, and as a reference only, according to Moreno [20], the average dietary energy consumption of Chile was around 2.200 kcal per person per day, slightly below our data for the 1930s.

Furthermore, the late 1970s were marked by an increase in poverty [26]. It is difficult to reconcile this fact with a supposed complete eradication of malnutrition. In 1977, Fernando Monckeberg, one of the most prominent experts in malnutrition in Chile, declared that undernutrition remained a serious concern for the country [23]. Figure 6 shows that it was only from the 1990s that the Chileans dietary energy consumption increased to above 2.700 kcal per person per day, reaching around 3.000 kcal during the last few years. Before the 1990s, effective social policies concerned with health and education made a greater impact on the reduction of stunting than a better diet. The nutrition transition was completed comparatively late in Chile, despite Núñez and Pérez claims that it gathered momentum from the 1930s. We have shown elsewhere that the per capita meat consumption stagnated between the 1930s and 1980s, but it increased markedly from the 1990s [27] (see also Mendoza et al. [82], which shows that animal proteins became increasingly important within the Chilean diet from the 1990s). According to FAO [83], as late as in 1990–1992, the proportion of undernourished people within the total population was still 8% in Chile. The decrease in child mortality during the 1970s and 1980s was the highest in Chilean history, at least if compared with the preceding decades [25], which is in line with our idea that there is room for improvements in malnutrition.

**Figure 7:**
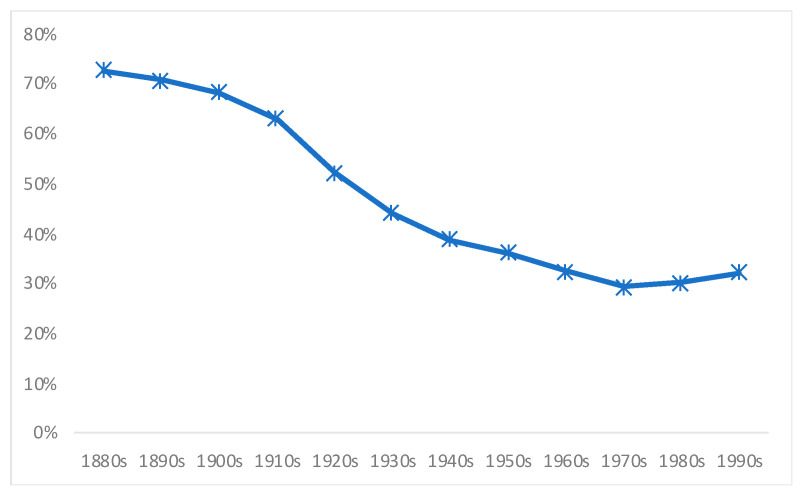
Chile’s poverty rates (share of the population). Source: Own elaboration from Prados de La Escosura [84].

Another important variable is the poverty rate: Those from low SES are more likely to be food insecure and to suffer infectious diseases, while diseases themselves prevent a better nutrition [34]. Figure 7 shows Chile’s average poverty rates per decade, and the similarities with our estimates of stunting rates for the whole twentieth century are striking. The reduction of the stunting rates operates in a similar fashion to the poverty rate. It is also telling that despite the remarkable improvements in reducing malnutrition, poverty remained an issue during the 1970s and 1980s. Writing in the late 1970s, Solimano and Hakim [70] made the crucial point that Chile’s main nutritional programs were closely linked to those children which were served by the educational and the health services. However, these services did not have universal coverage (i.e., they excluded the poorest of all), in order that the eradication of malnutrition could not be fully achieved.

Moreover, linked to poverty, child labour was not fully eradicated from the country until the late 1990s. As late as 1978, a new legislation had to be passed to ensure the abolition of child labour for those younger than 14, but it was still legal to employ youngsters aged 14–17 [60]. Finally, it is worth stressing that the improvement in stunting rates shown by our estimates during the 1980s and 1990s is in line with uninterrupted gains in life expectancy in Chile during these decades [6], with continuous improvements in mortality rates and general advances in health care [25], as well as with increases in real wages from the mid-1980s [20,79]. Even during the 1980s, and despite the economic crisis experienced by the country, the military government decided to focalize its social public spending on inexpensive programs aimed at improving the health and nutrition of the very poor (delivering feeding products with a high content of proteins and vitamins, including milk). This was an extreme focalisation of public social expenditure relying on the infrastructure accumulated during the welfare state period [26], as well as on the National Council for Feeding and Nutrition (CONPAN) created by Pinochet’s regimen itself [23].

## 6. Conclusions

Using a novel methodology, recently proposed by Salvatore [1], we have provided the first long-run estimates of stunting in Chile for cohorts born from the 1870s to the 1990s, for each decade. Therefore, we are making an important contribution to the study of Chile’s biological welfare in the long run, with particular reference to those belonging to the lower strata of society. Although we are aware that there is much to refine regarding this methodology, our results are consistent with alternative evidence, thus endorsing Salvatore’s method. As a result, it is worth highlighting the main trends. We found that stunting rates were very high during the last decades of the nineteenth century and early decades of the twentieth century, and that these rates increased during most of this early period, further supporting the idea of a deterioration in biological welfare in Chile during the late nineteenth century, despite both booming exports and economic growth [5,29]. These estimates are backed by alternative evidence for living conditions between circa the 1870s and 1910s: Very high infant (and general) mortality rates, a poor diet characterised by a low intake of animal proteins and dairy products, and generalised child labour. This is of consequence, since according to Fogel, it seems that “for the nineteenth century the biomedical measures are more laden with economic information than the traditional economic measures” ([30], p. 36).

Thereafter, despite the usual fluctuations of any long-term series, there was a clear downward trend from the 1910s to the 1990s. The gradual decrease in stunting rates from the 1910s was due to a combination of factors, whose importance varied over time, namely: Improved health (most noticeably seen in a sharp and continuous decline in both the infant and general mortality rates during the whole period); increased energy consumption (from a gradual rise in the 1930s to a substantial increase during the 1990s); a decline in poverty rates (in particular, between the 1930s and 1970s); and a reduction in child labour from the 1920s (although this is harder to quantify).

The emergence of a welfare state from the mid-1920s was a turning point in the development of public policies to tackle malnutrition. However, the Chilean state had already started to consider malnutrition as a national problem, and evolved national programmes to tackle it, as well as supporting private initiatives (as it did in many other areas of health and education). The first sanitary code of the country was promulgated in the late 1910s, while milk programs started even earlier, in 1902, although with little coverage. However, it was in the 1920s that the Chilean state intensified efforts to combat malnutrition, launching nationwide measures to improve the nutrition and health of the Chilean population, including the formal training of nutritionists from the 1930s, although the seeds of this development were planted two decades earlier.

Our new evidence provides further support to the idea that Chilean standards of living improved significantly during the period of industrialisation led by the state. The increased life expectancy of Chileans, and the low mortality rates among the elderly nowadays [25] may be linked to the diminution of malnutrition 60–80 years earlier, as Deaton suggested with reference to developed countries [32]. In addition, it conforms to the idea that the nutritional status affects the time of death [7]. Although it is true that during this period the per capita social expenditure increased, it is also the case that Chile remained a middle-income country, where it did not converge. The degree of success Chile achieved in diminishing malnutrition rates suggests that even for middle-income countries it is possible to reduce stunting rates significantly by applying the right nutrition policies at the right time. In Deaton’s words: The Chilean case shows that “there are ways of ensuring good health at low incomes” ([32], p. 44). This finding will be significant well beyond the national scope, since it may also explain reductions in contemporary health inequality in adults born when malnutrition rates were already low in Chile. This article should inform future public policies that aim to improve the biological welfare of the lower strata of society in developing countries.

Yet, despite these undeniable improvements, moderate and severe stunting were not fully eradicated in Chile during the period covered by this study, not even by the 1990s. The persistence of some social evils persisted: A delayed nutrition transition (i.e., only from the 1990s did the Chilean dietary energy consumption increase above 2.700 kcal per person per day); persistent inequality in both health and education [25,85]; income inequality and poverty; and even some child labour. We expect other scholars to follow Salvatore and our own studies, to be able to perform comparative studies between Argentina, Chile, and the rest of the Latin American countries for which there is comparable evidence. Furthermore, we expect colleagues to produce similar data but for females, although unfortunately, height data is often unavailable for long-term analysis.

## Figures and Tables

**Table 1:** Estimates of stunting rates in Chile, male adults born during the 1870–1990s.

Decade of Birth	Stunted at WHO 2007 Standard, HAZ < −2SD, Cases	Total Observations (Aged 18–55)	Stunting Rate at WHO 2007 Standard (%)
1870s	44	294	15.0%
1880s	101	576	17.5%
1890s	302	1.672	18.1%
1900s	713	3.676	19.4%
1910s	531	4.021	13.2%
1920s	229	2.656	8.6%
1930s	127	2.230	5.7%
1940s	285	3.354	8.5%
1950s	719	10.142	7.1%
1960s	43	1.616	2.7%
1970s	116	2.142	5.4%
1980s	124	3.702	3.3%
1990s	48	2.227	2.2%
Total	3.382	38.308	8.8%

Note: WHO 2007 standard: Mean of 176.1 cm and SD of 7.47 cm. Source: Own elaboration, data from military records, taken from [5,6].

## Data Availability

Data sharing not applicable.

## Appendix A

Evolution of Moderate and Severe Stunting in Chile (as a Share of Male Population, %), per Decade of Birth: Army Records vs. Navy Records, 1870–1890s.
